# Royal College of Surgeons of England

**Published:** 1844-08-24

**Authors:** 


### RECORD OF MEDICAL SCIENCE.

### ROYAL COLLEGE OF SURGEONS OF ENGLAND.

It is officially announced by the Royal College of Surgeons, that the following are the hospitals and schools of surgery and medicine in foreign countries, from which certificates of the professional education of candidates for the fellowship will be received by the college for the year commencing the 1st inst.

"The several hospitals and schools in the following cities, viz. Paris, Montpelier, Strasburg, Berlin, Vienna, Heidelberg, Bonn, Göttingen, Leyden, Pavia, New York and Philadelphia.

By order of the Council,
Edmund Belfour, Secretary."

July 13, 1844.

*London Lancet*, Aug. 3, 1844.

